# Fish Cytolysins in All Their Complexity

**DOI:** 10.3390/toxins13120877

**Published:** 2021-12-09

**Authors:** Fabiana V. Campos, Helena B. Fiorotti, Juliana B. Coitinho, Suely G. Figueiredo

**Affiliations:** 1 Centro de Ciências da Saúde, Departamento de Ciências Fisiológicas, Universidade Federal do Espírito Santo (UFES), Av. Marechal Campos 1468, Vitória 29040-090, Brazil; fabiana.v.campos@ufes.br (F.V.C.); helena.fiorotti@esib.butantan.gov.br (H.B.F.); juliana.b.goncalves@ufes.br (J.B.C.); 2 Laboratório de Bioquímica, Instituto Butantan, Av. Vital Brasil 1500, São Paulo 05503-900, Brazil

**Keywords:** fish venoms, cytolysins, multifunctionality, pore formation

## Abstract

The majority of the effects observed upon envenomation by scorpaenoid fish species can be reproduced by the cytolysins present in their venoms. Fish cytolysins are multifunctional proteins that elicit lethal, cytolytic, cardiovascular, inflammatory, nociceptive, and neuromuscular activities, representing a novel class of protein toxins. These large proteins (MW 150–320 kDa) are composed by two different subunits, termed α and β, with about 700 amino acid residues each, being usually active in oligomeric form. There is a high degree of similarity between the primary sequences of cytolysins from different fish species. This suggests these molecules share similar mechanisms of action, which, at least regarding the cytolytic activity, has been proved to involve pore formation. Although the remaining components of fish venoms have interesting biological activities, fish cytolysins stand out because of their multifunctional nature and their ability to reproduce the main events of envenomation on their own. Considerable knowledge about fish cytolysins has been accumulated over the years, although there remains much to be unveiled. In this review, we compiled and compared the current information on the biochemical aspects and pharmacological activities of fish cytolysins, going over their structures, activities, mechanisms of action, and perspectives for the future.

## 1. Introduction

Hundreds of venomous ray-finned fish species (Actinopterygii) are distributed in tropical and temperate marine and freshwater environments worldwide, being commonly associated with human injuries. Although this significant biodiversity translates into a promising source of bioactive compounds, the study of fish venoms is rather underrepresented in the literature [1]. Nevertheless, the number of accidents caused by venomous fish is by no means small, and some can have serious consequences and even be fatal [2,3].

**Figure 1:**
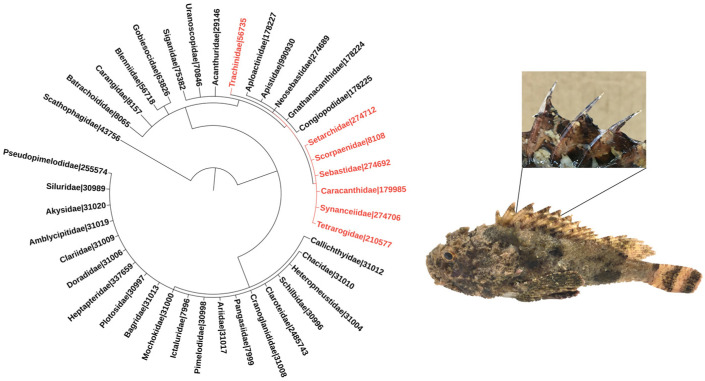
Phylogeny of venomous ray-finned fish families. The families included in the tree were listed based on the minimum estimate of venomous species according to [1]. Families belonging to the order Scorpaeniformes are highlighted in red, and so is the family Trachinidae from the order Perciformes. Numbers refer to NCBI taxonomic IDs. The phylogenetic tree was generated using the Interactive Tree of Life (iTOL–https://itol.embl.de; last accessed on 3 November 2021) tool [6]. The images adjacent to the phylogenetic tree depict a specimen of the scorpionfish *Scorpaena plumieri*, a member of the Scorpaenidae family, zooming in its dorsal venomous spines.

Venomous fish species belonging to the order Scorpaeniformes, which includes the families Synanceiidae (e.g., stonefish and devil stinger), Scorpaenidae (e.g., scorpionfish and lionfish), and Tetrarogidae (e.g., waspfish) [4] have been frequently associated with envenomation events [3]. Weeverfish from the family Trachinidae, order Perciformes, are also worthy of note [5] (Figure 1).

The venom apparatus of these fish is similar, and so are the biological activities associated with their venoms. The apparatus invariably consists of mucous-covered spines that can be located in different regions of the fish, being more common in the dorsal region (Figure 1), although several species have pectoral fins modified as a venom injection system. The secretory system is found in the anterolateral cavities of the spines, and may comprise well-defined glands or a set of more primitive, specialized secretory cells. Envenomation occurs when the victim threads or touches the spines, tearing the mucous sheath by mechanical pressure and causing the venom to be released into the wounds [7,8,9].

Excruciating pain is the most prevalent symptom of envenomation by fish, although local inflammation and a number of systemic responses, of which the most severe are cardiovascular and respiratory disturbances, are also observed [3,9,10]. Although envenomation by fish is rarely fatal to humans, under certain experimental conditions fish venoms are invariably lethal to animals [11,12,13,14,15].

Fish use their venoms mostly for defensive purposes, and the molecular composition of these venoms—combined with the evolution of strategies, such as mimicry and aposematism—reflects this fact [16]. Several bioactive compounds, including enzymes, such as hyaluronidases and proteases, large proteinaceous toxins, lectins, and peptides, among others, have been identified in fish venoms [17,18]. However, these venoms are somewhat unique in the sense that the biological activities underlying the major symptoms of envenomation are all induced by members of a high-molecular weighted protein family present in these venoms.

When first described, these extremely labile toxic components were referred to as “lethal factors” [12,19]. Physio-pharmacological studies on these lethal factors confirmed their multifunctional nature. These toxins reproduce the pain, inflammation, neurotoxic, myotoxic, and cardiotoxic effects induced by the crude venoms (for review, see [20,21]). In spite of this multifunctionality, the lethal activity associated with these toxins is attributed mainly to cardiac collapse and respiratory arrest [15,19,22].

In addition to the aforementioned effects, the multifunctional lethal factors also show strong hemolytic activity in vitro—a common feature in scorpaenoid fish venoms [13,19,23,24,25,26,27,28]; therefore, they are more accurately called membrane-damaging toxins or cytolysins [25]. A few cytolysins have been successfully identified in fish venoms and had some of their structural and functional features described (Table 1).

The wide spectrum of biological activities displayed by fish cytolysins implies that they play a major role in the envenomation process, which makes them subjects worthy of investigation. In this review, we will present a detailed overview of the biochemical and functional features of the known fish cytolysins. These toxins will be compared as to their structural and functional differences and similarities, and what has been put forward so far regarding their mechanisms of action will be discussed.

## 2. The Search for the Lethal Factor: Identification and/or Purification of Fish Cytolysins

Fish venoms can be collected through different methods, which may vary depending on the presence of defined glands or more primitive secretory systems. For those with defined glands—e.g., stonefish—the venom can be collected through puncture of the gland after removal of the tegument [46]. For those with primitive secretory cells—e.g., scorpionfish—the venom is usually obtained through the batch method, in which the spines are stripped and immersed in buffered solution, or the aspiration method, in which the spines are stripped and the venom aspirated from the grooves [44]. More recent methods, such as the sponge-in-a-tube method [47], in which a microtube containing a sponge is pressed against the spine to rupture the tegument and collect the venom, have the advantage of not requiring the removal of the spines and the sacrifice of specimens.

The search for a lethal factor in fish venoms started with the study of stonefish species, the most venomous of them all. The nature of the molecule responsible for the lethal activity associated with the venom of *Synanceia horrida* was first glimpsed in 1961, when it was found that only one of the seven protein fractions obtained through starch gel electrophoresis of the venom contained the heat-labile lethal material [46]. This fraction was almost twice as lethal as the crude venom when injected intravenously (i.v.) into mice tails (LD_50_: 18 versus 30 μg of nitrogen/kg). Ten years later, the lethal activity of the venom of the scorpionfish *Scorpaena guttata* was associated to a semi-purified instable fraction, which was almost three times as lethal as the crude venom (LD_50_: 0.9 versus 2.8 mg/kg, i.v.) [44]. The extreme lability of these fractions hindered the establishment of isolation processes, representing a major bottleneck for their research, which remained stagnant for many years.

By the end of the 20th century, when some of the difficulties imposed by the aforesaid lability of fish cytolysins were overcome through the establishment of proper purification and storage conditions [13,25,41,43], the biochemical and pharmacological characterization of these toxins was greatly accelerated. In addition, the combination of different methods, including liquid chromatography, cDNA cloning, automated Edman degradation, mass spectrometry (MS), and X-ray crystallography resulted in significant advances in the biochemical characterization of these molecules.

Cytolysins have been successfully purified from fish venoms through chromatographic steps using gel filtration, ion exchange, hydrophobic interaction, or adsorption, combined or not with fractionation by saline precipitation. Hemolytic and/or lethal activities were usually employed as a way of tracking the success of the purification process of these proteins [11,12,13,19,32,39,41].

From the venoms of the stonefish *S. horrida* and *Synanceia trachynis*, were purified stonustoxin (SNTX) [12] and trachynilysin (TLY) [32], respectively. The native molecular weights of SNTX and TLY were estimated in 148 kDa and 158 kDa, respectively. Both are heterodimeric proteins composed of two distinct subunits, named α and β, with masses of 71 kDa and 79 kDa for SNTX, and 76 kDa and 83 kDa for TLY. SNTX accounted for 9% of the protein content of the crude venom and was 22 times more lethal to mice (LD_50_ 0.017 μg/g; i.v.) [12,25,32].

Two different cytolysins, verrucotoxin (VTX) and neoverrucotoxin (neoVTX), were isolated from the venom of the reef stonefish *Synanceia verrucosa* [19,39]. VTX is a 320-kDa glycoprotein that in native form is organized in a tetrameric scaffold comprising two 83-kDa α and two 78-kDa β subunits [19]. It was twice as potent as the crude venom, being immediately lethal to mice at less than 60 ng/g (i.v.). NeoVTX, on the other hand, is a dimeric 166-KDa protein that comprises two distinct subunits (75-kDa α and 80-kDa β) and lacks carbohydrate moieties, showing structural features comparable to those of SNTX and TLY, while considerably differing from VTX [39]. It was also lethal to mice, with an LD_50_ of 47 μg/kg (i.v.).

The venom of the spotted scorpionfish *Scorpaena plumieri*, although considerably less harmful to humans than that of stonefish, also contains a lethal factor, named *S. plumieri* cytolytic toxin (Sp-CTx), which was first purified with very low yields (1%) [41]. Sp-CTx was then shown to be 12.3-fold more hemolytic (EC_50_ 56 ng/mL) than the crude venom. A molecular mass of 121 kDa was estimated for the native Sp-CTx, while a mass of 65,251 Da *m/z* was revealed by its mass spectrum, pointing to it having a dimeric nature with subunits of very similar molecular masses, much like SNTX and TLY [41]. However, unlike TLY and SNTX, Sp-CTx is a glycoprotein, displaying typical N- and O- linked glycoconjugate residues [41,43]. Three years later, the same group optimized the purification protocol of Sp-CTx and this new method increased the final yield by 13-fold when compared to the previous one [42]. The dimeric nature of Sp-CTx was then confirmed by cross-linking studies using bis-(sulfosuccinimidyl) suberate (BS3). Although lethality was not directly assessed in either of these previous reports, Sp-CTx was proved to be lethal when experiments conducted on anesthetized rats revealed that the animals eventually died upon receiving 70 μg/kg (i.v.) of the toxin [15].

In addition to the aforementioned toxins—which are the best chemically and functionally characterized fish cytolysins—a few such molecules have been purified from the venoms of other fish species.

From the lesser (*Echiichthys vipera*—also known as *Trachinus vipera*) and greater (*Trachinus* *draco*) weeverfish venoms were purified the cytolysins trachinine [11] and dracotoxin [13], respectively. Trachinine was purified by preparative electrophoresis and shown to be a 324-kDa molecule of tetrameric nature (81-kDa subunits), similar to VTX. It showed an LD_100_ of ˂ 100 μg/kg in mice (i.v.) [11]. Dracotoxin, unlike all the other known fish cytolysins, is a monomeric protein with MW estimated in 105 kDa both by SDS-PAGE and gel filtration. It was purified with high yields (36%) and it was lethal to mice (minimum lethal dose of 11 μg/g; i.v.) and ~20-fold more hemolytic than the crude venom of *T. draco* [13].

Semi-purified hemolytic fractions were obtained from lionfish (*Pterois lunulata*), devil stinger (*Inimicus japonicus*), and waspfish (*Hypodytes rubripinnis*) species. Based on the data obtained through gel filtration, immunoblotting, and cDNA cloning, these toxins were determined to be 160-kDa heterodimers composed of 80-kDa α and β subunits [40].

In spite of some variation as to the oligomeric functional state assumed by fish cytolysins purified so far, they are all high-molecular-mass proteins that appear to be active when assembled into oligomers, which could explain their extreme lability. Regarding the quaternary scaffold of these toxins, it is worthy of note that those described as dimeric had their native masses estimated by gel filtration chromatography, while, incidentally, those deemed tetrameric had their masses estimated by gel electrophoresis using the method described by [48].

Lethal and hemolytic activities have also been described in the venoms of the lionfish species *Pterois antennata* and *Pterois volitans* [14], although the actual molecules responsible for these activities have not yet been purified. Nevertheless, both venoms contain toxins identified through cDNA cloning and immunoblotting that share high sequence identity with stonefish cytolysins [14]. The venoms of the scorpaenoid fish species *Sebastapistes strongia*, *Scorpaenopsis oxycephala*, *Sebasticus marmoratus*, and *Dendrochirus zebra* also contain toxins, whose sequences were deduced from cDNA and genomic DNA data, that showed similarity with known fish cytolysins [45].

Finally, several studies have reported antigenic cross-reactivity between fish venoms. The antivenom raised against the venom of *S. trachynis* (SFAV)—the only commercially available fish antivenom—is effective in neutralizing not only the in vivo and in vitro effects of *S. trachynis* venom, but also those of other fish venoms [24,49,50,51]. In addition, it has been shown that the cytolysins isolated so far cross-react with SFAV and their effects can be neutralized by this antivenom [41,51], suggesting a close similarity between these molecules.

## 3. Analyzing the Structural Features of Fish Cytolysins

Before comparing and discussing the biological activities of different fish cytolysins, we will describe what is known so far regarding their structures. This overview should provide a useful background for a better understanding of such activities, particularly regarding the hemolytic activity associated with these toxins.

**Figure 2:**
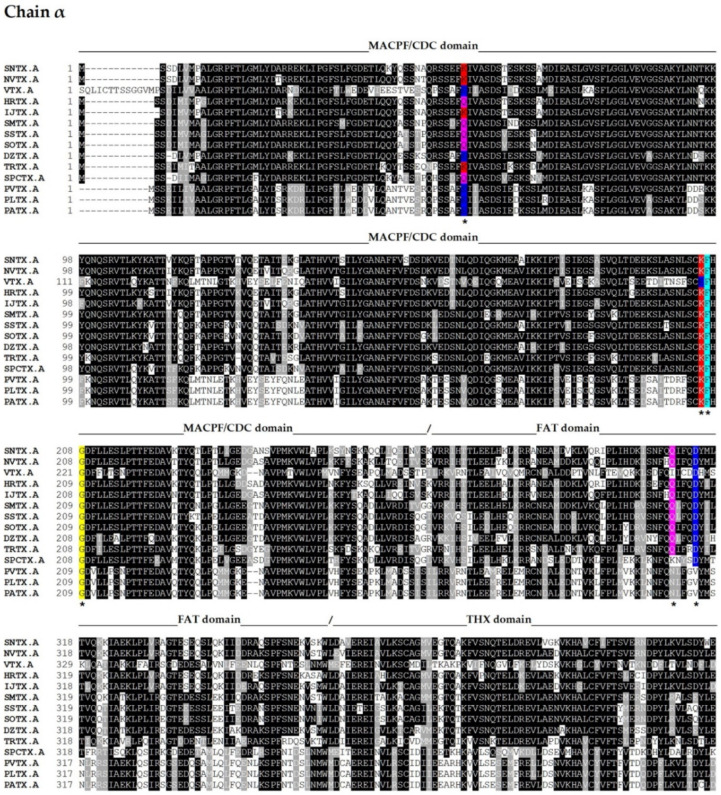

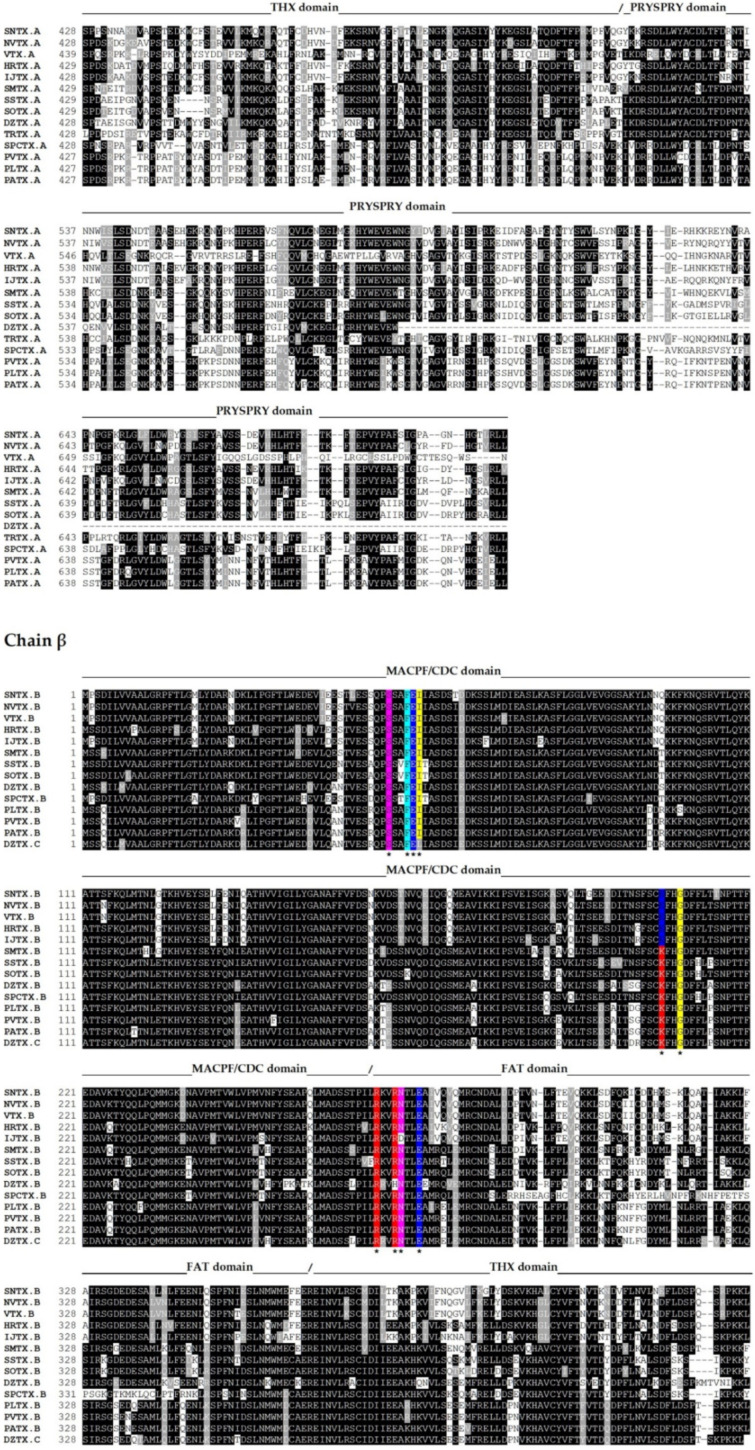

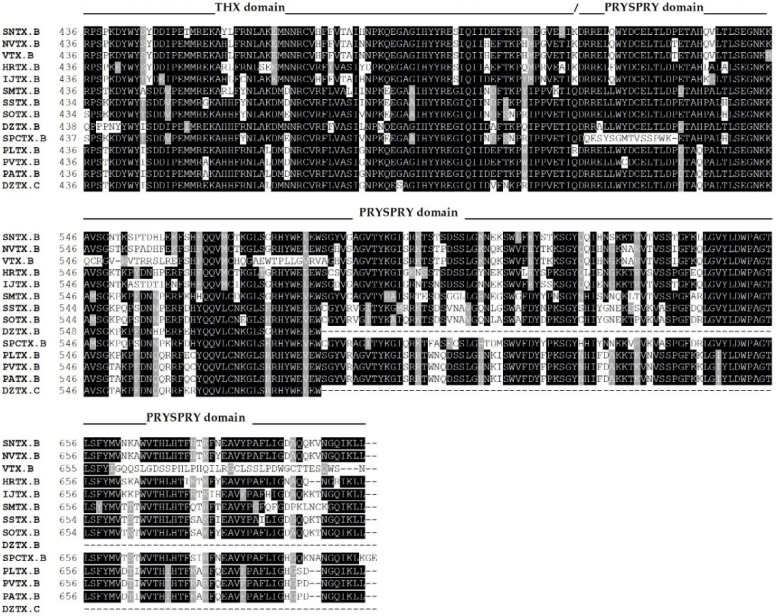
Primary sequence alignment of α and β chains of the cytolysins SNTX [A (Q98989); B (Q91453)] from *S. horrida*, VTX [A (CAA69254.1-partial); B (Q98993)] and neoVTX [A (A0ZSK3), B (A0ZSK4)] from *S. verrucosa*, HrTx [A (BAM74459.1); B (BAM74460.1)] from *H. rubriprinnis*, IjTx [A (BAM74457.1); B (BAM74458.1)] from *I. japonicus*, SmTx [A (AIC84040.1); B (AIC84041.1)] from *S. marmoratus*, SsTx [A (AIC84036.1); B (AIC84037.1)] from *S. strongia*, SoTx [A (AIC84038.1); B (AIC84039.1)] from *S. oxycephala*, DzTx [A (AIC84042.1–partial); B (AIC84043.1–partial)] from *D. zebra*, trachinine [A (AHY22717.1)] from *E. vipera*, Sp-CTx [A (A0A2P1BRQ0), B (A0A2P1BRP3)] from *S. plumieri*, PlTx [A (BAM74455.1); B (BAM74456.1)] from *P. lunulata*, PvTx [A (BAK18814.1); B (BAK18815.1)] from *P. volitans*, and PaTx [A (BAK18812.1); B (BAK18813.1)] from *P. antennata*. Alignments were performed on Muscle server. The sequence of dracotoxin from *T. draco* was not found. The species *S. trachynis* has been reclassified as a synonym of *S. horrida*, so that the sequence of TLY is reported as an alternative name for SNTX, under the same access code. Trachinine is referred to as echiitoxin on NCBI and only the α chain was found. Amino acid residues mentioned at the text are colored as yellow (nonpolar side chain); pink (polar side chain); blue (negatively charged side chain); red (positively charged side chain); and cyan (aromatic side chain) and marked with *. Domain marking was performed based on SNTX structure [55].

The complete amino acid sequences of several fish cytolysins were determined by cDNA cloning and/or genomic sequencing [13,14,39,40,45,52,53,54] (Figure 2).

These sequences, along with others totaling one hundred sequences (cover > 83%), were acquired by submitting the primary sequences of the α and β subunits of SNTX to the BLASTP algorithm [56] on the NCBI webserver (https://blast.ncbi.nlm.nih.gov/Blast.cgi; last accessed on 6 October 2021), employing the non-redundant protein sequences (nr) database from fish (taxid 7898).

**Figure 3:**
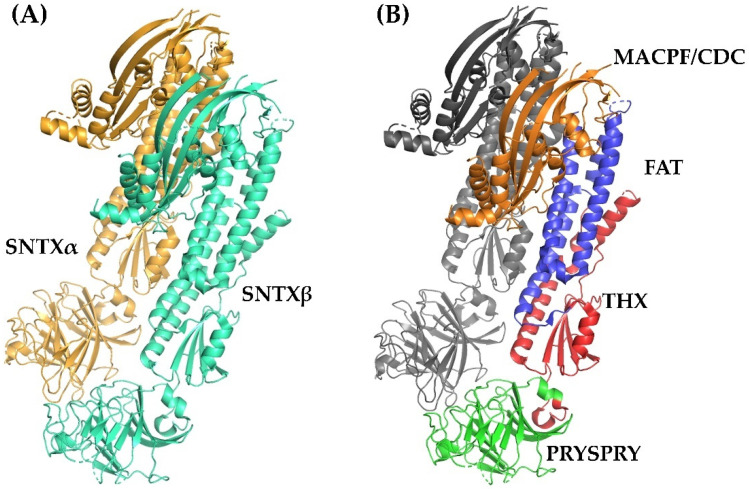
Crystallographic model of stonustoxin (SNTX). (**A**)—SNTX heterodimer showing the α (SNTXA-orange) and β (SNTXB-green) chains. (**B**)—SNTX domains: N-terminal MACPF/CDC domain (residues 1–265-orange); FAT domain (residues 266-385-blue); THX domain (residues 386-517-red), and C-terminal PRISPY domain (residues 518-703-green). Figure produced on Pymol using the model 4wvm deposited in the Protein Data Bank [55].

Fish cytolysins share high primary sequence similarity, with identity ranging from 45 to 94% and ~20% of residues present at conserved positions (Figure 2), suggesting a strong structural correlation between these molecules. However, only SNTX had its three-dimensional structure determined [55]. The crystallographic model (3.1 Å resolution) of SNTX (Figure 3A) showed that the α and β chains form a stable dimer with an extensive parallel interface along its longitudinal axis, maintained through polar interactions, such as hydrogen bonds and electrostatic interactions.

Fold recognition searches [55] showed that SNTX belongs to a branch of the “perforin-like” superfamily and revealed the presence of four domains (Figure 2 and Figure 3B) in each chain: (i) N-terminal MACPF/CDC domain (residues 1–265), homologous to the Membrane Attack Complex -Perforin/Cholesterol-Dependent Cytolysin domain. This domain is found in several species and represents a superfamily of pore-forming toxins; (ii) FAT domain (residues 266–385), with high structural similarity to the focal adhesion-targeting domain of human kinase-1. This domain is also found in several proteins and plays a role in the assembly of signaling complexes; (iii) THX domain (residues 386–517), with high structural similarity with thioredoxin-3 (THX-3) from *Saccharomyces cerevisiae*; and (iv) C-terminal domain (residues 518–703), which is similar to the PRYSPRY (PRY SPla and the RYanodine Receptor) domain, member of the tripartite motif family (TRIM) that participates in immune recognition in intracellular bacteria and viruses.

The sequences of the other fish cytolysins were analyzed using the Conserved Domains tool of the NCBI server (https://www.ncbi.nlm.nih.gov/Structure/cdd/wrpsb.cgi; last accessed on 6 October 2021) (Figure 2). Much like SNTX, they all showed the presence of the C-terminal SPRY (residues from 500 to 700, approximately) and the THX (residues from 380 to 500, approximately) domains on each subunit.

The similarity between these cytolysins and SNTX allows us to infer that some of the features described for the latter are shared among them all. For instance, the THX domain—which is usually associated with redox reactions—most likely plays a structural role, as the key cysteines of the THX catalytic motif are not conserved in these molecules. In addition, there appears to be electrostatic complementarity between the α and β chains through highly conserved charged residues (D314 and Q310 in SNTX-α; R269, N273, R272, and E276 in SNTX-β), which most likely participate in the polar interactions that take place in the interface between the chains. These interactions can be disrupted under the conditions used in purification and mass estimation processes, which may also account for the differences observed in the quaternary arrangements of fish cytolysins. Finally, four cysteine residues are conserved in all cytolysins analyzed, pointing to a possible role in heterodimer stability through disulfide bridges. However, the formation of these bonds was not observed in the three-dimensional structure of SNTX [55].

## 4. The Ubiquitous Hemolytic Activity of Fish Cytolysins: From the Definition of the Pore-Forming Mechanism to its Structural Confirmation

The cytolytic activity of almost all fish cytolysins isolated so far has been extensively characterized against erythrocytes, hence their being referred to as hemolysins in some instances. In spite of some divergence in the literature as to the potency and the species—specificity of the hemolytic activity of fish cytolysins, which can by at least partly explained by different experimental conditions—they are all most potent against rabbit erythrocytes [13,24,25], although the reason for such marked sensitivity remains unknown. In addition, a weak hemolytic activity against rat, mice, and cattle erythrocytes has been reported for some fish cytolysins, while those from human, sheep, pig, and chicken appear to be resistant to the lytic activity of these toxins [12,13,25].

There is also some evidence as to the lytic activity of fish cytolysins against other cell types. For instance, the prolonged exposure of neuro-glioblastoma NG108-15 cells to TLY led to an irreversible increase in membrane permeability [57]. In addition, SNTX lysed the membranes of platelets in rabbit blood in a dose-dependent way [58].

Cytolytic activity is often associated with the action of enzymes such as proteases and phospholipases and, in some cases, of C-type lectins such as CEL-III from the sea cucumber *Cucumaria echinata* [59,60]. However, fish venoms lack phospholipase activity [24,26,27,61,62,63] and, although enzymatic activity such as proteolytic and hyaluronidase has been described in these venoms, no such activity was associated with the purified lethal/hemolytic factors [12]. As to C-type lectins, although such molecules have been described in fish venoms [64], they are not associated with the hemolytic activity induced by these venoms [65]. Therefore, fish cytolysins were very soon believed to lyse erythrocytes through a direct, non-enzymatic mechanism [26].

The formation of non-selective transmembrane pores was soon shown to be the mechanism by which fish cytolysins destroy cells [42,57,66]. The pore-forming activities of SNTX and Sp-CTx were demonstrated through osmotic assays, in which the kinetics of the hemolysis was evaluated in the presence of osmotic protectants of different sizes [42,66]. While the smaller compounds raffinose and saccharose failed to prevent hemolysis, polyethylene glycols (PEGs) of ~1000–2000 reduced the rate of hemolysis induced by the toxins and PEGs > 3000 conferred almost full protection against it [42,66]. The pores formed by SNTX in the membrane of rabbit erythrocytes were estimated to be 2.5–3.2 nm in diameter [66].

The search for what mediates the interaction between fish cytolysins and the cell membrane began as soon as the first such toxins were purified. For instance, the hemolytic activity of the *T. draco* venom was found to be preceded by the binding of its hemolytic component to a protein receptor (glycophorin) on the surface of erythrocytes [13]. From then on, it became clear that cationic amino acid residues present in fish cytolysins are essential for their interaction with anionic, neutral or zwitterionic lipids in the membrane of erythrocytes, eventually leading to hemolysis.

In 1997, it was observed that SNTX no longer induced hemolysis when positively charged lysine and arginine residues in its surface were chemically modified, although the toxin’s secondary structure remained unaffected [66]. The modification of cationic residues also inhibited the lethal activity associated with SNTX [67]. These data are in agreement with structural motifs observed in these toxins, for instance, an amphiphilic, ~20-residues long α-helix flanked by regions rich in basic residues was predicted in both α- and β-subunits of SNTX, and it is believed to be the cationic site crucial for the toxin’s hemolytic activity [52].

As expected, the hemolytic activity of SNTX and neoVTX against rats and rabbit erythrocytes, respectively, was competitively inhibited by negatively charged lipids, most potently by cardiolipin and less so by phosphatidylserine and the gangliosides GM1 and GM2 [39,66]. Although cardiolipin was the most potent inhibitor, it is not present in the erythrocyte membrane, thus the hemolytic activity must be actually triggered by the electrostatic interaction between the toxins’ cationic residues and anionic phospholipids such as phosphatidylserine, which is abundant in the membrane of erythrocytes [39]. It stands to reason that the species-specificity of the hemolytic activity of fish cytolysins could be related to the density of these lipids in the erythrocyte membrane.

The electrostatic interaction between anionic lipids and toxins is influenced by the number of negative charges and the pKa of acidic groups in the lipids, as indicated by the different inhibitory potencies of different lipids [66]. This conclusion is further supported by the fact that the maximal hemolytic activity of Sp-CTx is achieved between pH 8 and 9 [43]. Although the neutral lipids phosphatidylcholine, phosphatidylethanolamine, sphingomyelin, and cholesterol did not affect the hemolytic activity of SNTX and neoVTX [39,66], that which was induced by Sp-CTx in rabbit erythrocytes was inhibited by phosphatidylethanolamine, phosphatidylglycerol, and cholesterol [43]. Similar results regarding the role of differently charged lipids on the hemolytic activity were found for the venomous extract of the lionfish *P. volitans* [68], which is yet another indication that this venom contains cytolysins homologous to those identified in other fish species.

The presence of the MACPF/CDC domain in fish cytolysins [53,55] and the inhibitory effect of cholesterol on the hemolytic activity of Sp-CTx [43] suggest that this lipid is also required for the interaction between these toxins and the erythrocyte membrane, much like the MACPF/CDC proteins found in bacteria.

The hemolytic activity of fish cytolysins is influenced by factors other than the cationic residues present in their surfaces. That of Sp-CTx, for instance, is calcium dependent, being abolished by zinc ions [43]. Furthermore, five of the 15 cysteine residues and 10 of the 18 cysteine residues of SNTX and neoVTX, respectively, are free, and these free thiol groups, much like the tryptophan residues in the surface of SNTX, also play an important role in the hemolytic activity induced by these toxins [39,52,69].

**Figure 4:**
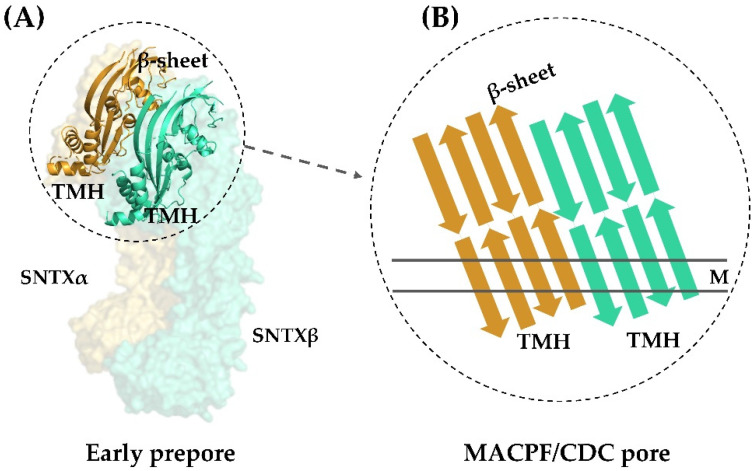
Transmembrane pore formation mechanism. The SNTX heterodimer represents an early and soluble phase of the pore formation mechanism (**A**). After the interaction between chains α and β, the MACPF/CDC domain helices undergo a conformational change to form the continuous β-sheet of the transmembrane pore (**B**). (**A**) was produced on Pymol using the model 4wvm deposited in the Protein Data Bank [55] and (**B**) was adapted from [55]. TMH—transmembrane helices; M—membrane.

All this evidence pointing to a pore-formation mechanism being responsible for the hemolytic activity of fish cytolysins was corroborated by the three-dimensional structure of SNTX. Pore-formation was shown to be the result of an interaction between the α- and β-chains along their longitudinal axis through charge complementarity between the MACPF/CDC domains. These domains—which are formed by twisted four-stranded antiparallel β-sheets with two adjacent bundles of α-helices (transmembrane helices—TMH)—interact on the heterodimer to form a soluble early pre-pore. After that, the TMH from each chain unwind to form a large, continuous β-sheet that comprises the β-barrel of the pore (Figure 4).

This arrangement is in agreement with the complementarity between MACPF/CDC monomers described for other perforins, which directs initial oligomerization events (pre-pore assembly). In SNTX, this pre-pore contains 20 SNTX subunits (or 10 SNTX-α/β heterodimers) that line up along a horizontal plane [55].

Furthermore, the PRYSPRY domain was shown to be responsible for the initial interaction of SNTX with the cell surface. Consistent with this function, this domain is located in the solvent-exposed face of SNTX, in a position analogous to the Ig and C2 domains of CDC and perforins, which mediate protein–protein and protein–lipid interactions in the recognition of pathogens by TRIM immune proteins [55].

The high sequence similarity and the presence of conserved domains in the other fish cytolysins identified so far allows us to predict that these proteins perform their cytolytic functions through the same mechanism. For instance, the residues G208 (α-chain) and G209 (β-chain) of SNTX, reported as necessary for pre-pore formation, are present in all cytolysins. So are the residues F206 (α-chain) and F54, I56, and S52 (β-chain), related to the formation of hydrogen bonds between the β4-strand of SNTX-α and the β1-strand of SNTX-β, which are also involved in pre-pore formation (Figure 3).

Moreover, the pairs K205 (α-chain)-E55 (β-chain) and K54 (α-chain)-E206 (β-chain) present in SNTX’s β4 (α-chain) and β1 (β-chain) strands form ionic pairs analogous to a partially closed zipper, which contributes to pore formation. These ionic pairs are conserved in almost all cytolysins, in spite of exchanges as to which residue belongs to each chain.

In addition to the structural evidence, the formation of large ring-shaped pores was visualized in rabbit erythrocytes by transmission electron microscopy (outer dimension: 257 ± 5.7 Å (mean ± SEM); lumen i.d.: 117 ± 4.5 Å) [55], in agreement with what had been predicted for the pores formed by SNTX and Sp-CTx [42,66].

## 5. Pharmacological Activities Associated to the Multifunctional Fish Cytolysins

### 5.1. Cardiovascular Activity

The most potent and best-studied pharmacological activity associated with the venoms of Scorpaeniformes is that on the cardiovascular system [9,23,28]. The profound cardiovascular alterations induced by these venoms are attributed to the action of cytolysins, being closely related to the lethality of these toxins [15,19,22].

The molecular mechanisms underlying the cardiovascular effects induced in vivo and in vitro by cytolysins from the venoms of stonefish and scorpionfish have been fairly well documented. Despite some variability, which could simply reflect the fact that different studies employed different doses and experimental models, it is a consensus that these toxins cause drastic changes in blood pressure, which are the result of their effects on the blood vessels and heart.

When the purification of VTX from the venom of *S. verrucosa* was first described, it was observed that the injection of this cytolysin (~16 μg/kg, i.v.) into anesthetized rats induced an immediate hypotensive effect that evolved to cardiac arrest [19]. A drastic, irrecoverable fall in the blood pressure of anesthetized rats has also been described for SNTX (20 μg/kg, i.v.) and Sp-CTx (70 μg/kg, i.v.), although a biphasic response characterized by an increase followed by a decrease on arterial pressure was observed in these instances [15,22].

Consistent with the hypotension observed in vivo, a significant vasorelaxation has been associated with the action of fish cytolysins on vascular preparations [22,29,30,41]. In addition, a biphasic response on rat aortic rings pre-contracted with phenylephrine, characterized by an endothelium- and dose-dependent relaxation followed by a contractile phase, was associated with both SNTX and Sp-CTx, albeit with different intensities. Regardless, the fact that both toxins induced relaxation of isolated aortic rings in the picomolar range (68 pM and 1 pM for SNTX and Sp-CTx, respectively), attests to the high hypotensive potency of these molecules [22,29,41].

The vasorelaxant phase of the effect induced by SNTX and Sp-CTx was irreversibly abolished by 25 and 10 μM of the nitric oxide (NO) synthesis inhibitor N^G^-nitro-L-arginine methyl ester (L-NAME), respectively [22,41]. In addition, the relaxant response associated with SNTX was reversibly inhibited by the guanylate cyclase inhibitors methylene blue (10 μM) and hemoglobin (5 mM) [22]. It was also reduced by the H_2_S inhibitors DL-propargylglycine (PAG; 1 mM) and β-cyano-L-alanine (BCA; 1 mM), being potentiated when these inhibitors were individually combined with L-NAME (1 μM) [30]. The K^+^ channel blocker tetraethylammonium (TEA; 3 mM) and the substance P (SP) receptor antagonist N-acetyl-L-tryptophan-3,5-bis(trifluoromethyl)-benzyl ester (NATB; 0.5 mM) also reduced the SNTX-induced vasorelaxation, which was completely blocked by the iNOS inhibitor AMT-HCl (0.5 mM) [29].

Based on these results, it has been proposed that SNTX acts by binding directly to the SP receptor in the endothelial cell or by promoting SP release. The consequent increase in intracellular [Ca^2+^] stimulates iNOS and increases the production of NO, which will be released by the endothelial cell and absorbed by the smooth muscle cell. There, it increases the levels of cGMP, which will then positively modulate K^+^ channels, hyperpolarizing the membrane and causing relaxation [29]. The SNTX-induced relaxation also involves the synergistic action of H_2_S and NO [30]. The high sequence identity and structural similarities between fish cytolysins, as well as the antigenic cross-reactivity displayed by these toxins [14,40,41], suggest that the aforementioned mechanism of action can be extended to cytolysins other than SNTX.

The effect of fish cytolysins on the heart itself has also been explored to a certain extent. In 1997, the cardiotoxicity of the freshly purified but unstable VTX (1 μg/mL) and an impure but more stable fraction named p-VTX (0.001–1 μg/mL) was assessed [36]. Both exerted negative inotropic and chronotropic effects on the peak contraction and accelerated the relaxation phase in frog atrial fibers [36]. The negative inotropic effect induced by p-VTX was reduced in higher external [Ca^2+^], while the chronotropic effect remained unaffected in these conditions. Moreover, p-VTX hyperpolarized the fibers and decreased the duration of both the plateau and the hyperpolarizing phase of action potentials recorded in these fibers. Both muscular and electrical activities of the atrial fibers was counteracted by glibenclamide, an ATP-sensitive K^+^ channel (K_ATP_) blocker. The nature of these responses suggests p-VTX acts by inhibiting Ca^2+^ influx into the fibers, possibly by negatively modulating Ca^2+^ channels, and by positively modulating K_ATP_ channels.

On the other hand, studies conducted in guinea pig ventricular myocytes, employing electrophysiology and various pharmacological strategies, showed that, unlike what had been observed in atrial fibers, VTX inhibits K_ATP_ currents via the activation of the muscarinic M3 receptor-PKC pathway and increases L-type Ca^2+^ currents by activating the β-adrenergic-cAMP-PKA pathway [37,38]. As pointed out by [37], this discrepancy regarding the effects of VTX on frog atrial fibers and guinea pig ventricular cells could be simply the consequence of different species having different cardiac regulatory mechanisms. Accordingly, TLY (1 μg/mL) acted much like VTX in frog atrial fibers, hyperpolarizing the membrane, shortening the action potentials, and inducing a negative inotropic effect, in addition to a contracture due to acetylcholine release. However, this negative inotropic effect associated with TLY, although Ca^2+^-dependent, was not blocked by Cd^2+^, indicating it does not involve voltage-dependent Ca^2+^ channels [33].

In addition to its effects in vivo and on isolated vessels, Sp-CTx had its cardiotoxic activity assessed in rat isolated hearts and papillary muscles [15]. The toxin (10^−9^ to 5 × 10^−6^ M) induced a transient, concentration-dependent positive inotropic effect, characterized by a drastic increase in left ventricular pressure. It also induced vasoconstriction on the coronary bed by increasing vascular perfusion pressure. The biphasic pattern described in anesthetized rats and rat aortic rings [15,41] was not observed in isolated hearts.

In isolated papillary muscle, Sp-CTx (0.1 μM) increased myocardial contractility through a pathway involving the activation of β-adrenoreceptors, as the pre-treatment with the β-blocker propranolol and the catecholamine releasing agent tyramine inhibited this response [15]. Post-rest contraction experiments suggested that the cardiac response induced by Sp-CTx involves an increase in sarcolemmal Ca^2+^ influx, which was corroborated by the reversible 18% increase in L-type Ca^2+^ currents induced by 1 nM of this toxin in isolated ventricular myocytes [15]. Taken together, these results point to Sp-CTx acting by increasing Ca^2+^ influx into cardiac cells via modulation of L-type Ca^2+^ channels through the activation of the β-adrenergic signaling pathway, much like what had been described for VTX in guinea pig ventricular myocytes [38].

It has been suggested that the pore-forming mechanism underlying the hemolytic activity of fish cytolysins might also account for some of the other pharmacological activities associated with these toxins [22,55,57,66]. However, the aforementioned studies showing that SNTX, TLY, VTX, and Sp-CTx mediate their cardiovascular effects through complex pathways involving the release of vasorelaxant mediators and the modulation of membrane receptors and/or ion channels [15,33,36,37,38], point to pore-formation not being the underlying cause of these responses. It is also important to stress that, although seemingly conflicting depending on the experimental conditions, these pathways could actually contribute in a synergistic way to potentiate the cardiovascular toxicity attributed to these cytolysins.

### 5.2. Neuromuscular Activity

In addition to the cardiovascular effects induced by fish venoms, symptoms observed after envenomation, such as weakness, muscle spasms, and even paralysis, are an evident sign of neurotoxicity. Indeed, under experimental conditions, the crude venoms of *S. verrucosa, S. horrida (trachynis), P. volitans*, and *T. draco* have had their neurotoxic effects demonstrated [13,70,71,72].

At least for the estuarine stonefish venom, the neuromuscular activity could be associated directly to its cytolysins—SNTX and TLY. In 1994, the effects of SNTX (8 to 50 µg/mL) on the neuromuscular function were investigated in mouse and chick skeletal muscles in vitro and rat skeletal muscle in vivo [31]. SNTX produced an irreversible, concentration- and time-dependent block of nerve- and muscle-evoked twitches of the mouse hemidiaphragm. Similar effects were observed in the chick biventer cervicis muscle, in which nerve-evoked twitches and the contractures induced by acetylcholine (200 µM), carbachol (8 µM), and KCl (40 mM) were blocked by SNTX (22 and 44 µg/mL). The ability of SNTX to block muscle-evoked twitches, as well as the marked blockade of this effect by dantrolene sodium (6 µM)—a muscle relaxant that inhibits Ca^2+^ release from intracellular stores—but not by the nicotinic receptor antagonist tubocurarine (15 µM), suggested that SNTX acts directly on the muscle to inhibit neuromuscular function, inducing contractures via a Ca^2+^-dependent rather than an acetylcholine-dependent mechanism. In addition, SNTX did not block conduction in the frog sciatic nerve, which also points to the conclusion that the toxin affects neuromuscular function via myotoxicity [31].

On the other hand, TLY—which had its neuromuscular activities rather well explored—was reported to act presynaptically by causing the release and depletion of neurotransmitters from nerve terminals [32], as had been previously shown for the crude venom itself [71]. This cytolysin (2 to 20 µg/mL) irreversibly increased the frequency of miniature end plate potentials in frog cutaneous pectoris preparations, indicating it stimulated the quantal release of acetylcholine [32]. A similar outcome was found in *Torpedo marmorata* neuromuscular junction, in which TLY (30 to 60 nM) increased the spontaneous quantal neurotransmitter release in an irreversible way [34].

This neurosecretory role attributed to TLY was supported by the observation that botulinum toxin—a neurotoxin that disturbs exocytosis—inhibits the release of catecholamines induced by this cytolysin (60 nM) in bovine adrenal chromaffin cells [35]. However, unlike in motor nerve endings, where TLY depleted the number of small vesicles almost completely (86%) without affecting that of large-dense core vesicles [32], in chromaffin cells, this cytolysin mediated a soluble N-ethylmaleimide-sensitive fusion protein attachment protein receptor (SNARE)-dependent release of catecholamines from large core vesicles [35].

As expected, this neurosecretory effect was Ca^2+^-dependent, although voltage-gated Ca^2+^ channels do not seem to participate in the process, as the specific inhibition of various Ca^2+^ channel isoforms failed to affect this response in both neuromuscular junction and chromaffin cells [34,35]. Nevertheless, fluorescence-based evidence showed that TLY does promote a transient influx of Ca^2+^ into chromaffin cells in some other way, and its effect on neurosecretion was in fact blocked by the non-specific inhibitor La^3+^. In addition, the toxin induced a more sustained, localized increase in intracellular Ca^2+^ levels through release from caffeine-sensitive intracellular stores [35].

Further evidence as to how TLY affects membrane permeability came to light in 2002, when the voltage-clamp technique was used to investigate the mechanism of action underlying the neurosecretory effects induced by this cytolysin [57]. The perfusion of neuroblastoma/glioma cells (NG108-15) with TLY (12 nM) for ~1 min increases membrane conductance, by means of an inward cationic current that was inhibited by anti-TLY antibodies and La^3+^. A lack of ionic selectivity was confirmed by the current reversing around 0 mV. The rather complex nature of the macroscopic current induced by TLY, which comprises both distinct steps and low fluctuations, and the unusually high single channel conductance associated to the current steps, point to the target of TLY being other than pre-existing channels. In addition, TLY-induced currents were also recorded in membrane patches free from channels. Taken together, these results, and the fact that, once bound, the toxin cannot be washed out, indicate pore-formation as the most likely mechanism underlying the increase in conductance promoted by TLY in neuroblastoma/glioma cells [57]. The fact that the membrane must be exposed to a certain amount of toxin for some time in order to suffer permeability changes supports this conclusion, being in fact a requirement predicted by the pore-forming model proposed for SNTX [55,66].

### 5.3. Pain-Inducing and Inflammatory Activities

Immediate pain is the most distressing feature of envenomation by fish venoms, being disproportionate to the resulting wound in the absence of notable secondary tissue injury. In fact, counteracting pain is usually the main focus of treatment when the envenomation does not progress to systemic complications. Some experimental evidence points to the participation of cytolysins in the pain as well as in the local inflammatory process that rapidly develops on the site of injury caused by fish venoms.

The first association between fish cytolysins and these local effects was made when SNTX was first isolated in 1991 [12]. It was then observed that the toxin (0.15–0.4 mg, intraplantar injection—i.pl.) induced a dose-dependent edema (a minimum edema dose of 0.15 µg) within 1 h of injection into mouse hind paws, which lasted for more than 24 h and could not be blocked by pre-treatment with the antihistamine diphenhydramine (50 mg/kg, intraperitoneal injection—i.p.). No reference was made then as to the possible nociceptive effects induced by SNTX.

On the other hand, nocitoxin, a mildly hemolytic toxin isolated from the venom of the bullrout *Nothestes robusta*, was deemed to be the pain-causing protein in this venom, as only the fraction containing it could reproduce the algesic effect induced by the crude venom in human subjects [27]. There is, however, some doubt as to its falling into the fish cytolysin category, as it did not cross-react with the stonefish antivenom.

The nociceptive and inflammatory activities of Sp-CTx have been relatively well described when compared to those of other fish cytolysins. The first indication that Sp-CTx was at least partly responsible for the pain and swelling induced by the venom of *S. plumieri* came from the fact that these responses were blocked by the stonefish antivenom, which, as previously mentioned, reacted against a protein with the biochemical characteristics of this toxin [51]. The confirmation came in 2018, when [43] showed that the injection of Sp-CTx into mouse hind paws (0.1–3 μg, i.pl.) caused a dose-dependent nociceptive response and an intense, sustained, and also dose-dependent edema response that reached its peak within 60 min and persisted for up to 4 days.

Further insights into the mechanism underlying the local inflammatory reaction induced by fish cytolysins were obtained when these same authors reported that the swelling induced by Sp-CTx was reduced by pre-treatment with the bradykinin B2 receptor antagonist HOE-140 (100 nm/kg, i.p.) or the serine-proteases inhibitor aprotinin (8 mg/kg, i.p.) [43]. These findings indicate that the kallikrein-kinin system participates in this response, as had been already proposed when the inflammatory activity of the crude venom of *S. plumieri* was investigated a few years earlier [73]. However, these drugs did not prevent the edema response completely [43], suggesting that additional pathways might contribute to the onset of the inflammatory response. Moreover, the nociceptive activity induced by Sp-CTx could not be blocked by either drug, which indicates that it does not share the same mechanism described for the inflammatory activity [43].

That is, in fact, one of the biggest gaps in the study of fish venoms, and, by proxy, of fish cytolysins: the mechanisms behind the pain-inducing activity associated with these molecules remain largely unknown. It has been suggested that pore formation might be the underlying cause [43,55], but there is also evidence against this hypothesis. For instance, while investigating the pain caused by the lionfish *P. volitans* venom, [74] found that the heat-labile, protein components responsible for this activity appear to act specifically on neuropeptidergic nociceptors, although the identity of these components has yet to be determined.

## 6. Conclusions

Fish cytolysins stand out among other animal toxins because they alone can reproduce the main in vitro and in vivo effects induced by the venoms in which they are contained. These multifunctional toxins account for the extreme pain and inflammatory events experienced by the victims of envenomation by fish and affect the neuromuscular and cardiovascular systems in a significant way, potentially leading to death. Not to mention the species-specific hemolytic activity that, although seemingly irrelevant where envenomation is concerned, characterizes this type of toxin.

**Figure 5:**
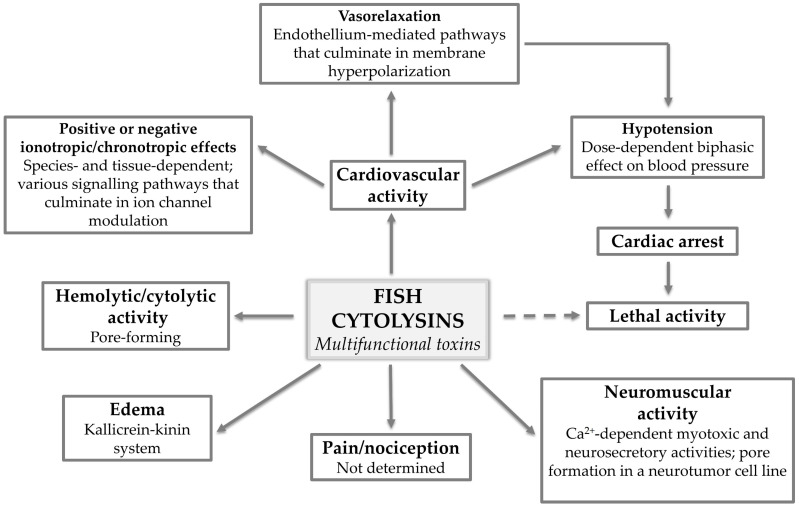
Fish cytolysins: multifunctional toxins. The various pharmacological activities displayed by multifunctional fish cytolysins and what has been determined regarding the mechanisms of action underlying each activity.

Although intriguing, this multifunctionality also adds to the complexity surrounding the mechanisms of action underlying the different activities displayed by these toxins (Figure 5). At this point, it is established that fish cytolysins destroy cells by forming non-selective pores in the membrane, but how much this pore-forming ability actually contributes to the pharmacological activities induced by these molecules is a matter for discussion.

Ample evidence points to fish cytolysins affecting the cardiovascular and neuromuscular systems through the modulation of signaling pathways that might vary in different tissues and species. However, that does not exclude the possibility that these toxins might also be able to form pores in the membrane of the cells that compose these systems. Based on the data gathered so far, we propose it to be a function of dose, time of exposure, and—naturally—the presence of proper recognition sites in the aforesaid membranes. Future studies on fish cytolysins should take this possibility into account.

All in all, much has been done regarding the investigation of biochemical and pharmacological features of fish cytolysins, considering how very labile and complex these molecules are. Nevertheless, as exposed in this review, there are still considerable gaps and contradictions, especially where what we believe to be their multi-mechanistic mode of action is concerned. The major role played by fish cytolysins in the envenomation process, added to the many questions raised by their multifunctionality, fully justifies the quest for a better understanding of how these molecules act.

## Figures and Tables

**Table 1:** Venomous fish and their cytolysins.

Family	Species	Cytolysin	Activity Described
Synanceiidae			
Estuarine stonefish	*Synanceia horrida*	SNTX *-Stonustoxin	Lethal [12] Hemolytic [12] Cardiovascular [22,29,30] Neuromuscular [31] Edematogenic [12]
	*Synanceia trachynis*	TLY *-Trachynilysin	Lethal [25] Hemolytic [32] Cardiovascular [33] Neuromuscular [32,34,35]
Reef stonefish	*Synanceia verrucosa*	VTX *-Verrucotoxin neoVTX *-Neoverrucotoxin	Lethal [19] Hemolytic [19] Cardiovascular [36,37,38] Lethal [39] Hemolytic [39]
Devil stinger	*Inimicus japonicus*	IjTx **-*I. japonicus* toxin	Hemolytic [40]
Scorpaenidae			
Scorpionfish	*Scorpaena plumieri*	Sp-CTx *-*S. plumieri* cytolytic toxin	Lethal [15] Hemolytic [41,42] ardiovascular [15,41,42] Edematogenic [43] Nociceptive [43]
	*Scorpaena guttata*	Unnamed toxin **	Lethal [44]
	*Sebastapistes strongia*	SsTx ***-*S. strongia* toxin [45]	-
	*Scorpaenopsis oxycephala*	SoTx ***-*S. oxycephala* toxin [45]	-
	*Dendrochirus zebra*	DzTx ***-*D. zebra* toxin [45]	-
	*Sebasticus marmoratus*	SmTx ***-*S. marmoratus* toxin [45]	-
Lionfish	*Pterois volitans*	PvTx ***-*P. volitans* toxin [14]	-
	*Pterois antennata*	PaTx ***-*P. antennata* toxin [14]	-
	*Pterois lunulata*	PlTx **-*P. lunulata* toxin	Hemolytic [40]
Trachinidae			
Greater weeverfish	*Trachinus draco*	Dracotoxin *	Lethal [13] Hemolytic [13]
Lesser weeverfish	*Echiichthys vipera #*	Trachinine *	Lethal [11]
Tetrarogidae			
Waspfish	*Hypodytes rubriprinnis*	HrTx **-*H. rubriprinnis* toxin	Hemolytic [40]

(*) purified protein; (**) semi purified lethal/hemolytic large protein; (***) identified at the cDNA level only; (#) also known as *Trachinus vipera*.

## Data Availability

Not applicable.
